# Supplementary material for: Proteomic Analysis of a Rice Mutant sd58 Possessing a Novel d1 Allele of Heterotrimeric G Protein Alpha Subunit (RGA1) in Salt Stress with a Focus on ROS Scavenging
Source: Int J Mol Sci. 2019 Jan 4;20(1):167. doi: 10.3390/ijms20010167 (PMC6337198; doi:10.3390/ijms20010167)
Supplement: Supplementary file 1 [file ijms-20-00167-s001.zip › RGA1-salt ijms supplementary materials 12.25.docx]

**Supplementary Materials**


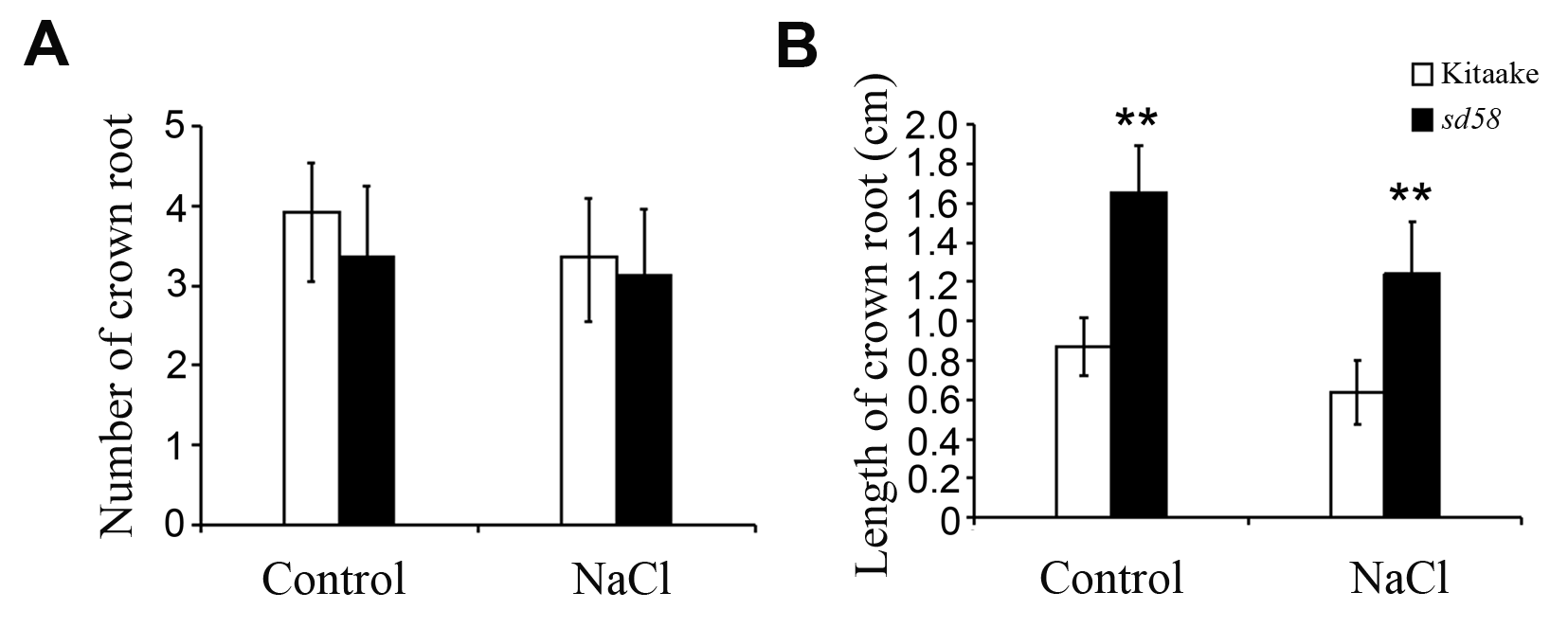


**Figure S1.** *RGA1* negatively regulates crown root development at seedlings stage. The number (A) and length (B) of crown root of 5-d-old Kitaake and *sd58* seedlings grown under 100 mmol/L NaCl after germination was measured. Values are means ± SD. Student’s *t*-tests were used to assess the significant differences between *sd58* and Kitaake plants under the same conditions (**p* < 0.05, ***p* < 0.01).


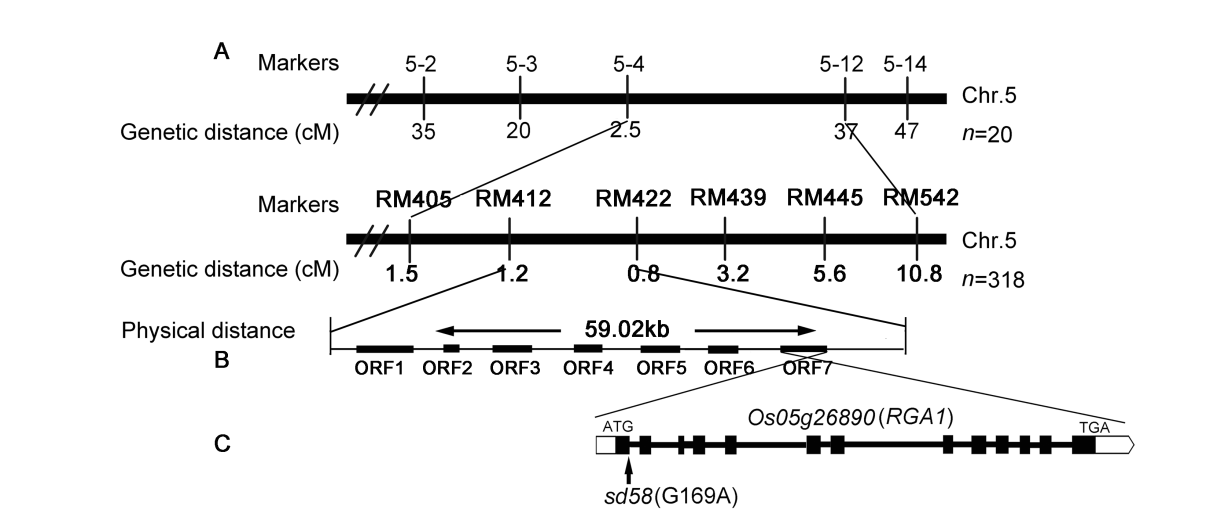


**Figure S2.** The positional cloning of *RGA1*. (A) *RGA1* was first found to be linked to the SSR (simple sequence repeat) marker on chromosome 5; fine mapping narrowed its location to a 59.02 kb genomic DNA segment flanked by insertion-deletion (InDel) markers RM412 and RM422; (B) the critical genomic region’s sequence includes 7 open reading frames; (C) the structure of the *RGA1* candidate gene LOC_Os05g48390. Both the start (ATG) and stop (TGA) codons are indicated. Black boxes indicate coding sequence. The mutant site in the *RGA1* sequence is shown.


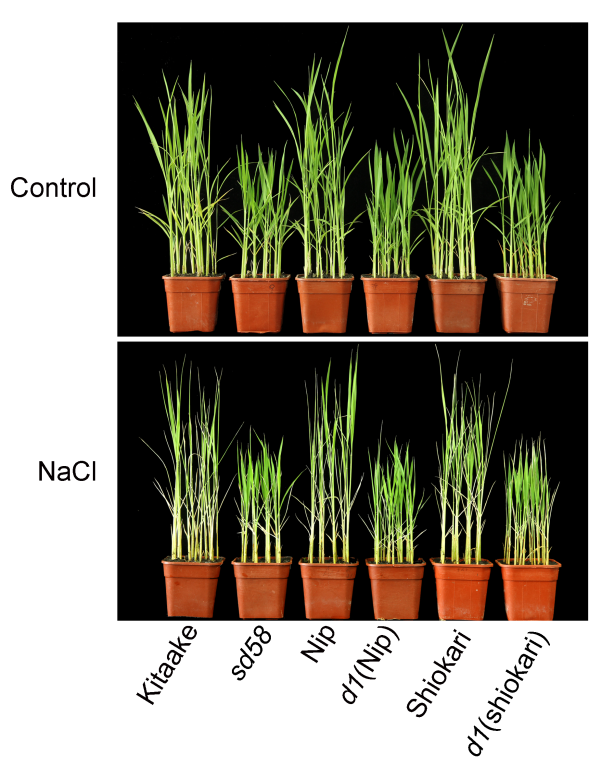


**Figure S3. *RGA1* negatively regulates salt tolerance in rice.** The salt tolerance phenotype of three allelic mutants of *RGA1* in different genetic background including Kitaake (*sd58*), Nipponbare (*d1*) and Shiokari (*d1*), respectively


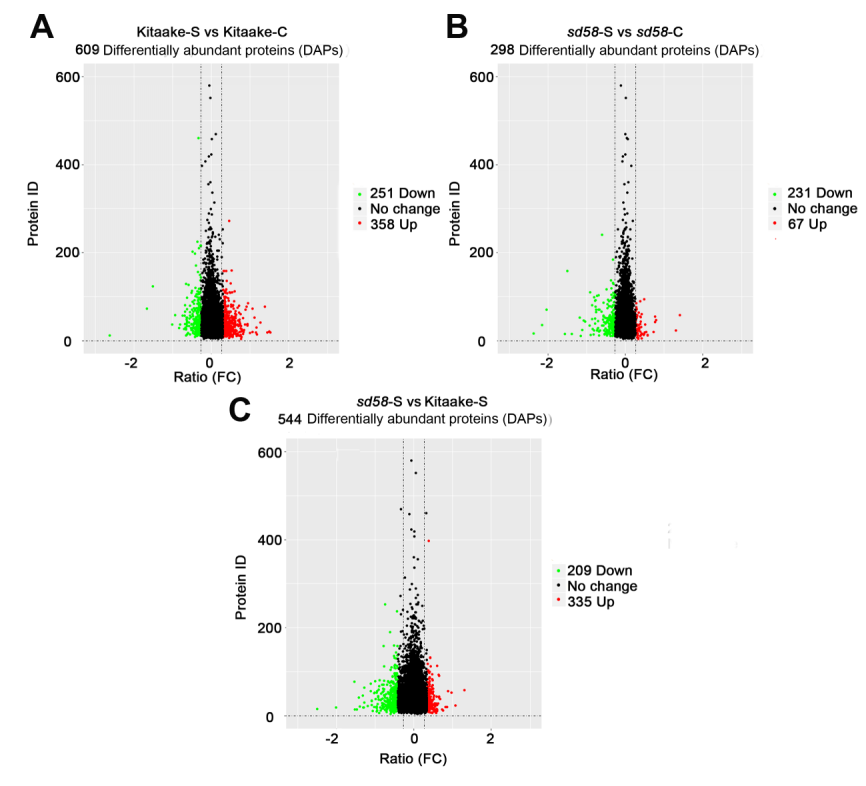


**Figure S4.** Ratio distributions for the identified differentially abundant proteins (DAPs). The changes in differentially abundant proteins (DAPs) of *sd58* and Kitaake in response to salt stress were analyzed. The horizontal axis displays ratios of Kitaake-salt vs Kitaake-control (A), *sd58*-salt vs *sd58*-control (B) and *sd58*-salt vs Kitaake-salt (C). The red point indicates that the ratio was greater than 1.2, and the green point indicates that the ratio was less than 0.83.


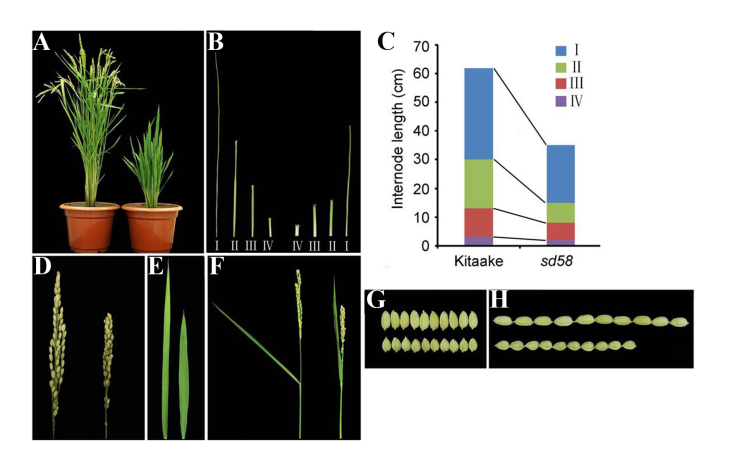


**Figure S5.** The agronomic characteristic phenotypes of the *sd58* mutant. Comparison of agronomic characteristic phenotypes between Kitaake (left or up panel in each part) and *sd58* mutant (right or down panel) on the mature plant height (A), internode length (B), panicle length and compactness (D), flag leaf (E), flag leaf angle (F), grain width (G) and grain length (H). (C) Statistics data of the internodes length.

**Table S6.** Performance of agronomic and morphological traits in the wild type and *sd58*

| **Traits** | **WT** | ***sd58*** |
| --- | --- | --- |
| Plant length (cm) | 69.74±2.92 | 43.38±2.22** |
| Panicle length (cm) | 13.20±1.23 | 8.79±0.71** |
| Effective tillering | 20.33±3.56 | 27.23±5.66** |
| Grain number per panicle | 59.00±8.22 | 60.93±7.73 |
| Filled grains per panicle | 55.70±7.56 | 33.33±6.20** |
| Grain length (cm) | 0.68±0.02 | 0.47±0.04** |
| Grain width (cm) | 0.33±0.02 | 0.34±0.02 |
| 1,000-grain weight (g) | 20.10±0.12 | 13.89±0.04** |
| Flag leaf angle (°) | 46.28±9.84 | 11.50±2.53** |
| Flag leaf width (cm) | 0.93±0.08 | 1.40±0.07** |

Data shown are mean ± SE from 20 plants. ** indicates significant difference at P < 0.01 level by Student’s t test.

**Table S7.** Primers used in this study.

| Primer | Forward | Reverse |
| --- | --- | --- |
| **Molecular markers used for gene mapping** | | |
| 5-2 | 5'-TCACACACTGACAGTCTGAC-3' | 5'-AATGTGGCACGTGAGGTAAG-3' |
| 5-3 | 5'-CATCCATACATTATACCACCACT-3' | 5'-GTAGCCCCTCACAAACAGAG-3' |
| 5-4 | 5'-CAGAAGCCTTCTTACGAACTT-3' | 5'-TAATCTTTGAGCGAGCAATT-3' |
| 5-12 | 5'-GCTCTCCTGTGGGTTTTCAG-3' | 5'-CATGGTGCTCCTACTGGTTG-3' |
| 5-14 | 5'-CCGTGTGTTTGTTGATAAATG-3' | 5'-CATATCAAAACCACCATGGTT-3' |
| RM18405 | 5'-GATCGAACCAGCGCCTTTATC-3' | 5'-GGTGGAGGAGGAAGCAACACC-3' |
| RM18412 | 5'-ACAACTGCCGATCGATTCAC-3' | 5'-GTATAGGTCCAAAGCTCACC-3' |
| RM18422 | 5'-AATACCCGTGCGTTGTACCG-3' | 5'-CTTCCATCGCCTCTTGCACG-3' |
| RM18439 | 5'-CGAAATGTGGCCAGCATTTG-3' | 5'-GATATTGATGTATTTCTAAA-3' |
| RM18445 | 5'-GGTTTCGCTGATTCCGTCTC-3' | 5'-CTTTAGTCCCGGATTGGTAC-3' |
| RM18452 | 5'-AGACAACTAGAGGTAGCACATCT-3' | 5'-GTCGGGATTAAATTCTCGCTAGG-3' |
| **Primers for Sequencing** | | |
| RGA1-g | 5'-GGATCCTGAGATCTAGACGT-3' | 5'-AGGAACAAAGTTTCACACTG-3' |
| RGA1-s1 | 5'-TCTTTACTCAACAGTTAAAG-3' |  |
| RGA1-s2 | 5'-CTGTCCATGTTCATAGACAA-3' |  |
| RGA1-s3 | 5'-GAGGTATATAGGTTGTATGA-3' |  |
| RGA1-RT | 5'-ATGGAGAGCTTCTTCGTCTT-3' | 5'-TTAATTATGAGAACTATGCACG-3' |
| PLB | 5'-CGACTCACTATAGGGAGAGCGGC-3' | 5'-AAGAACATCGATTTTCCATGGCAG-3' |
